# Computational design of functional random heteropolymers through atomistic simulations

**DOI:** 10.1371/journal.pone.0343799

**Published:** 2026-03-18

**Authors:** Tianyi Jin, Collin S. Lung, Ting Xu, Connor W. Coley, Alfredo Alexander-Katz

**Affiliations:** 1 Department of Chemical Engineering, Massachusetts Institute of Technology, Cambridge, Massachusetts, United States of America; 2 Department of Materials Science and Engineering, Massachusetts Institute of Technology, Cambridge, Massachusetts, United States of America; 3 Department of Materials Science and Engineering, University of California, Berkeley, Berkeley, California, United States of America; 4 Department of Chemistry, University of California, Berkeley, Berkeley, California, United States of America; 5 Materials Science Division, Lawrence Berkeley National Laboratory, Berkeley, California, United States of America; 6 Kavli Energy NanoScience Institute, University of California, Berkeley, California, United States of America; 7 Department of Electrical Engineering and Computer Science, Massachusetts Institute of Technology, Cambridge, Massachusetts, United States of America; Beni-Suef University, EGYPT

## Abstract

Random heteropolymers (RHPs) are emerging single-chain nanoparticles with great potential in protein mimicry, yet a systematic understanding of how chemical composition and monomer structures govern their structure, dynamics, and hydration remains limited. Using atomistic molecular dynamics simulations, we examine how various design parameters, including chain length, backbone architecture, charged monomer concentration, chain-level composition, and side-chain micropolarity influence RHP assembly and hydration behavior. As chain length increases, methacrylate-based RHPs transition from rod-like to random-walk statistics and ultimately collapse into compact globules stabilized by hydrophobic collapse and methacrylate-poly(ethylene glycol) (PEG) interactions. Positively charged monomers follow the Hofmeister series in their hydration. Interestingly, the dimerization results from hydrophobic and PEG-positively charged-monomer interactions, and not from opposite charge interactions. Alternative backbones such as acrylate and (meth)acrylamide display sequence-dependent compactness and dynamics, reflecting greater chemical sensitivity. PEG side-chain length strongly affects solubility and hydration, with shorter side chains making the overall chain more hydrophobic. Also, we show that branching-induced micropolarity modulates local hydration patterns of hydrophobic residues. Overall, these results establish general molecular design principles for tuning the assembly and dynamics of RHPs through compositional and chemical control, providing a foundation for engineering synthetic polymers that mimic the compactness, hydration, and functional adaptability of proteins.

## Introduction

Proteins are the functional workhorses of living organisms. By modifying their amino acid sequences, proteins can perform a wide array of tasks, facilitated by natural evolution and selection [1]. The precision and selectivity of proteins are rooted in the well-established sequence-structure-function paradigm [2,3]. To address these challenges, researchers have turned to synthetic alternatives that offer greater resilience, cost-effectiveness, and scalability. Compared to natural proteins, these synthetic alternatives can be designed and manufactured more efficiently while maintaining functional versatility, positioning them as practical platforms for applications where proteins face constraints in stability and large-scale production. The vast diversity of synthetic monomers, exceeding the 20 canonical amino acids, provides opportunities to develop functionalities that surpass those found in nature. A promising synthetic approach involves single-chain nanoparticles (SCNPs), which mimic versatile protein functions including catalysis and sensing [4–10]. SCNPs are synthesized using intramolecular cross-linking of polymer precursors with reactive pendant groups, enabling them to fold into nanosized, well-defined structures with specific functions. Examples of reactive pendant groups include moieties for metal binding [11,12] and organocatalyst [13–15]. Upon cross-linking, polymer scaffolds are accessible to substrates, yielding SCNPs capable of specialized enzyme-mimetic tasks.

Recently, methacrylate-based (MMA-based) RHPs have gained significant interest as a versatile family of SCNPs capable of mimicking multiple protein-like functions. RHPs, often referred to as random or statistical copolymers, consist of two or more monomers randomly distributed along a polymer chain. The hydrophobic MMA backbone, complemented by diverse side chains or pendant groups, collapses into molten globules with complex internal structures [16, 17]. We would like to highlight that in our design, the reactive groups responsible for specific functionalities are not pre-attached as pendant groups to the linear polymer precursors. Instead, the RHPs are engineered to create a tailored microenvironment that can subsequently independently recruit reactive groups, such as heme, after polymerization [18]. This modular approach enhances the versatility of RHPs, enabling the incorporation of diverse functional components without the constraints of predefined pendant group chemistries. Furthermore, using a similar design approach (without the recruitment of reactive groups), MMA-RHPs have demonstrated multiple protein-like functions beyond catalysis [19]. These include binding to proteins and preserving their functions in non-native environments [20–23], as well as transporting protons rapidly and selectively across lipid membranes [24,25]. Other functions of MMA-RHPs include the formation of membraneless organelles capable of encapsulating small organic molecules, providing a versatile platform for molecular encapsulation and compartmentalization [26,27], and serving as adhesive hydrogels [28]. The ability of MMA-based RHPs to interact with biomacromolecules, including DNA [23] and proteins [20,23], highlights their potential as delivery vehicles (*e.g.*, copolymer polyplexes [29–31]), as polyclonal antibody [32] and for enhancing enzyme functionality in industrial processes such as composting [21,33,34] and other harsh or non-native environments.

Unlike natural proteins, which rely on precise sequences and well-defined three-dimensional structures, RHPs employ a sequence ensemble and thus a structure ensemble to achieve designated functions through specific combinations of monomers and compositions. While RHPs may not achieve the same precision and selectivity as proteins, their collective behavior within an ensemble provides a population-based alternative that serves as a functionally versatile platform [23]. These functionalities are not only designable but also evolvable through the optimization of the physicochemical properties of the polymer, including chain length, monomer chemistry, and composition [23,35,36]. Additionally, functionality can be further refined through the optimization of RHP blend formulations, enabling synergistic interactions and enhanced performance [37].

We have developed a computational protocol to study the assembly of RHPs using atomistic molecular dynamics (MD) simulations in an explicit solvent [38]. This protocol includes an initial collapse in an implicit solvent followed by an annealing cycle in an explicit solvent, making it adaptable to RHPs with varying monomer identities and compositions. Using this approach, we have investigated the molecular fundamentals underlying protein-mimicry properties. MMA-based RHPs, formulated as reported in 2018 [20], collapse into compact globules with limited backbone mobility and highly mobile side chains [38,39]. These characteristics resemble the molten globule state of proteins [40,41]. The globules exhibit soft glass transitions and heterogeneous dynamics [39]. This glassy nature hinders chain reconfiguration in water [42] and at solid-liquid interfaces [43], while the globules can remodel in organic solvents such as DMSO, THF, and hexane [44] and at liquid-liquid interfaces [45]. Our simulations also reveal that these globules possess chemically heterogeneous surfaces, featuring both hydrophobic and hydrophilic groups [38] with a rugged energy landscape[46]. The molecular origin of this heterogeneous surface lies in hydration frustration, wherein hydrophobic groups are hydrated, and polar or hydrophilic groups are dehydrated. This phenomenon mirrors a key feature of protein functionality, including binding and catalysis [47,48]. The hydration frustration arises from the negative Flory-Huggins interaction parameter (*χ*) between the PMMA backbone and polar/hydrophilic polyethylene glycol (PEG) side chains [49]. Specifically, the PMMA backbone folds into a globule to minimize surface interactions, while the PEG pendant groups partition between the backbone globule and the aqueous solvent, leveraging their favorable interactions with both PMMA and water. Hydrophobic ethylhexyl groups, expected to form a core, are excluded from the center of the globule due to spatial occupancy by PEG moieties. This leads to the unexpected exposure of hydrophobic groups to water. The polarity of these groups plays a critical role in determining their hydration patterns and overall surface behavior. Notably, some protein-mimetic properties of RHPs, such as compactness, hydration, and chaperoning, are sequence-insensitive. This lack of dependence on precise sequence control suggests that one-pot synthesis is sufficient to achieve convergent functionalities in these RHPs [50]. These RHPs can laterally bind to *β*-barrel membrane proteins and stabilize them by interacting with their loop regions and reducing structural fluctuations [51]. This finding underscores the practicality and scalability of RHPs as synthetic platforms for mimicking protein-like behaviors [50].

In this work, we extend our previous studies to examine how subtle chemical variations in backbone architecture and side-chain chemistry influence the assembly and dynamics of RHPs in water. We specifically focus on the effects of chain length, the incorporation of positively charged monomers, and the dimerization behavior of MMA-based RHPs on hydration patterns that are critical to their protein-like properties. In addition, we investigate how backbone architecture and chain-level composition contribute to sequence-sensitive behaviors. Finally, we explore the role of side-chain micropolarity arising from variations in poly(ethylene glycol) length and the degree of branching, which strongly affects hydration through nuanced differences in partial charge distribution. We find that MMA-based RHPs exhibit distinct scaling and morphological behaviors as chain length increases. The incorporation of positively charged monomers in single-chain RHPs and RHP dimers has negligible electrostatic effects due to the glassy nature of the PMMA backbone. Hydration of these monomers follows the Hofmeister series, with even hydrophobic charges remaining well-hydrated, underscoring the difficulty of burying charges in these systems. MMA-based RHPs exhibit sequence-insensitive compactness and form frustrated globules, while peptoid- and peptide-based RHPs also exhibit sequence-insensitive compactness but form core-shell globules. In contrast, MA-, MAn-, and MMAn-based RHPs display sequence-sensitive compactness and form core-shell globules. RHP dynamics are similarly sensitive to sequence. In MAn-based RHPs, through a two-step chain-level composition evolution, hydration patterns are determined by the synergistic effect of chain-level composition and sequence identity, whereas compactness is dictated solely by chain-level composition. Furthermore, the length of the PEG pendant group strongly influences solubility, with shorter PEG chains exhibiting reduced hydration and increased hydrophobicity. The micropolarity difference in the side chains [e.g., linear vs. branched alkyl chain, and poly(propylene glycol) vs. poly(ethylene glycol)] is key to their hydration. Together, these findings highlight the tunable nature of RHPs through adjustments in monomer chemistry and composition. MMA-based backbones emerge as a robust platform for designing sequence-insensitive, compact globules, offering significant potential for applications as protein mimetics and beyond.

## Materials and methods

### System setup

Sequence ensembles with a degree of polymerization (DP) of 100 or 200 are generated using the Composition Drift Program [52,53], targeting a composition of 50% methyl methacrylate (MMA), 25% oligo(ethylene glycol) methacrylate (OEGMA) with nine PEG repeating units terminated with a methyl group, 20% 2-ethylhexyl methacrylate (EHMA), and 5% 3-sulfopropyl methacrylate (SPMA). SPMA is fully ionized as a strong acid in the experimentally relevant pH conditions that are close to physiological levels. From the generated sequence ensemble, ten sequences are randomly selected. The selected set of sequences has been shown to adequately represent the sequence space [50]. The sequences of 20mer and 50mer are selected from our previous work [39]. All sequences are shown in S1 Fig in S1 File. In systems containing positively charged monomers, 5% of the OEGMA monomers are randomly replaced with these positively charged monomers. Due to the stochastic nature of sequence generation, the overall composition of ten sequences and the chain-level compositions of individual sequence may slightly differ from the targeted composition. Chirality is assigned randomly when required to create a racemic mixture.

Two-component copolymer sequences are randomly generated. Two sequences, one with the smallest (SEQ1) and the other with the largest (SEQ2) radius of gyration (R_g_) of backbone atoms, are chosen as the starting points for chain-level composition evolution. These sequences are subsequently mutated in two steps by random substitution to match the chain-level composition of the other sequence.

The backbone architectures include poly(methyl methacrylate) (PMMA), poly(methyl acrylate) (PMA), poly(methyl acrylamide) (PMAn), poly(methyl methacrylamide) (PMMAn), peptide, and peptoid. The selected backbones span a broad range of hydrophobicity, solubility, and flexibility [54]. Peptide and peptoid backbones are included due to their established relevance in biomimetic design, while MA and MMA provide chemically distinct, experimentally accessible synthetic analogs that allow us to explore the influence of backbone chemistry on polymer behavior.

In summary, each system is defined by N = #sequences×#conformations. For 20mer, 50mer, and 100mer four-component MMA-based RHPs, N = 10×10 = 100 simulations are performed, while the 200mer system uses N = 10×1 = 10 simulations. Systems containing positively charged monomers and their dimers are simulated with N = 10×1 = 10. For MA-, MAn-, MMAn-, and peptide-based RHPs, N = 10×10 = 100, and for the peptoid-based RHP, N = 10×1 = 10. To examine the effects of sequence, two representative sequences are selected (SEQ1 and SEQ2) from MA-, MMA-, MAn, MMAn, and peptide-based RHPs, resulting in N = 10×2 = 20 simulations. For the two-component copolymer systems, N = 1×1 = 1 simulation is performed. The mutated sequence sets include N = 10×10 = 100 simulations. Simulations exploring PEG side-chain length variations use N = 10×10 = 100 for MMAn-based RHPs, while systems with octyl or Jeffamine-like side chains use N = 10×1 = 10. The error bars are calculated as the standard deviation across all N simulations.

### Molecular dynamics simulations

The General Amber Force Field (GAFF) is used [55]. Partial charges for all monomers are assigned using the restrained electrostatic potential (RESP) method [56], calculated with Gaussian 16 Revision C.01 [57]. This parameterization does not include explicit polarization and relies on Hartree-Fock–level calculations, but it has been extensively validated for homopolymer and copolymer systems in previous studies [38,42–45,49–51,58,59]. These results support the suitability of this parameterization for accurately capturing the structural and thermodynamic properties of the polymers studied.

Polymer parameterization and monomer assembly into extended chains are performed using AmberTools19 from Amber 18 [60]. Langevin thermostat with a collision frequency of 2 ps^-1^ is used for temperature control in all simulations [61]. Bond lengths involving hydrogen atoms are constrained using SHAKE algorithm [62]. Isotropic Berendsen barostat with a time constant of 1.0 ps is used for pressure control [63]. The simulations are performed with a 2 fs time step, using the molecular dynamics leapfrog integrator. Electrostatic interactions are calculated using the Particle Mesh Ewald (PME) method with a real-space cutoff of 8.0 Å [64]. The van der Waals interaction cutoff is also set to 8.0 Å.

The simulation protocol follows our previous work [38] and generally consists of two stages: (1) collapse of an initially extended linear chain in an implicit solvent, and (2) subsequent annealing and equilibration in an explicit solvent. The polymer system, prepared using AmberTools 19, first undergoes a brief energy minimization using the steepest descent method, transitioning to the conjugate gradient descent method after 1000 cycles. It is then minimized using the generalized Born/surface area implicit solvent model [65]. The system is then heated to 500 K over 18 ps, maintained at this temperature for 2 ps, and equilibrated at 500 K for 20 ns. The annealing protocol involves cooling the system from 500 K to 300 K over 6 ns, followed by a 74-ns hold at 300 K. No periodic boundary conditions are applied during the implicit solvent simulations. The resulting conformation obtained is explicitly solvated using the SPC/E water model, with potassium or chloride counter ions added to neutralize the system, using monovalent ion parameters from [66]. The choice of the SPC/E water model provides the highest accuracy in reproducing bulk water dynamics and structure compared with other common models such as TIP3P and SPC [67].

The solvated system undergoes a second minimization step, again using the steepest descent method transitioning to the conjugate gradient descent method after 1000 cycles. It is then heated to 300 K over 18 ps and maintained at this temperature for 2 ps in an NVT ensemble. Following this, the system undergoes a 2-ns equilibration at 300 K and 1.0 bar. An NVT annealing cycle is then conducted, consisting of 20 ns at 650 K, 40 ns of cooling from 650 K to 300 K, and a final 20-ns equilibration at 300 K. This annealing process facilitates relaxation of the polymer structure in the explicit solvent. The system is subsequently maintained at 300 K and 1.0 bar for 40 ns, which serves as the production simulation used for analysis. In our previous work, we have shown that the structures are metastable due to the glassy nature of the system, while physicochemical properties such as size and monomer hydration equilibrate within 40 ns [38,49,50]. Periodic boundary conditions are applied in all directions.

For each five-component RHP, a dimer system is examined, consisting of two identical RHP conformations extracted from the final frame of the production run, using PACKMOL [68]. In the initial structures, one pair of oppositely charged monomers from the two chains is intentionally positioned in close proximity to examine specific charge-pair interactions. The same simulation protocol as the single-chain RHP system is applied, except that no annealing runs are performed.

Trajectory and data analysis are performed using AmberTools19 and Python packages (*numpy* and *pytraj*), with latter interfacing with Amber’s *cpptraj* program [69,70]. Data are represented as all data points, the median, the lower and upper quartiles, and whiskers extending within 1.5 times the interquartile range (IQR).

The **spatial distance between monomers [R(s)]** are calculated for C*α* atoms, and the **backbone radius of gyration (R**_**g**_**)** are determined based on all backbone atoms. The **asphericity** is calculated using the formula:


$$Asphericity=\frac{(I_{1}-I_{2}{)}^{2}+(I_{2}-I_{3}{)}^{2}+(I_{1}-I_{3}{)}^{2}}{\frac{1}{2}(I_{1}+I_{2}+I_{3}{)}^{2}}$$ (1)

where I_1_, I_2_, and I_3_ are the principal moments of inertia of the structure. The value of asphericity ranges from 0 (spherical) to 1 (rod-like).

**Density** is calculated using the mass of the sequence and the estimated volume derived from R_g_. The population of water molecules in the first solvation shell (FSS) is determined separately for each monomer and normalized by their maximum values to obtain the **normalized first solvation shell (NFSS)**. The atomic **root-mean-square fluctuations (RMSF)** of backbone atoms and the standard deviation of the backbone dihedral angles (**SD dihedral angle**) are calculated over the last 5 ns of the production runs. For example, a 20mer MMA-based RHP contains 20 backbone atoms and 37 backbone dihedral angles.

The sequence sensitivity is assessed based on convex hull analysis described in our previous work [50]. For each conformation, the dimension of monomer hydration, represented by NFSS values, is 73, corresponding to 42 methyl groups, 14 ethylhexyl groups, 14 ethylene glycol groups, and 3 sulfopropyl groups, which are common across all the sequences. The convex hull in the space pf principal components 1 and 2 (PC1 and PC2) is defined as the smallest convex set enclosing those, where each point represents one conformation of the same sequence.

## Results and discussions

### Effect of chain length on four-component MMA-based RHPs

**Fig 1:**
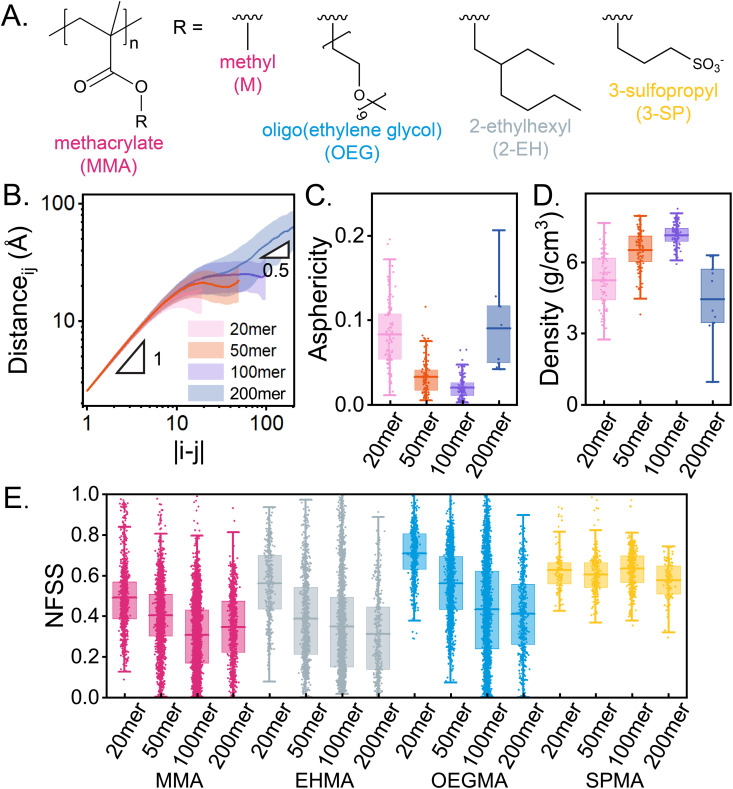
Effect of chain length on four-component MMA-based RHPs. (A) The chemical structure of MMA-based RHPs, with monomers color-coded as follows: MMA in magenta, OEGMA in blue, EHMA in gray, and SPMA in yellow. Side chains are abbreviated as “M”, “OEG”, “2-EH”, and “3-SP”, respectively. (B) The spatial distance between monomer i and monomer j [R(s), s=|i−j|] for RHPs with degrees of polymerization (DP) of 20 (N = 100, pink), 50 (N = 100, orange), 100 (N = 100, purple), and 200 (N = 20, navy). Insets show the slopes for the low-s and high-s regimes. Error bars indicate standard deviations across all RHP conformations. (C) Asphericity and (D) density of RHPs across four selected chain lengths. **(E)** NFSS for each monomer type in RHPs across four chain lengths. Data are represented as all data points, the median, the lower and upper quartiles, and whiskers extending within 1.5 times the interquartile range (IQR).

The starting point of this work builds on the design of four-component biomimetic MMA-based RHPs, as reported in 2018 [20]. These polymers consist of 50% MMA (short or backbone monomer), 25% OEGMA (polar monomer), 20% EHMA (hydrophobic monomer), and 5% SPMA (negatively charged monomer) (Fig 1A). Here, we examine the assembly of RHPs with degrees of polymerization (DP) of 20, 50, 100, and 200, with sequence schematics shown in S1 Fig in S1 File. Previous works have demonstrated that 20mer, 50mer, and 100mer RHPs collapse with distinct metastable states [38,39,42]. The RHP structures are described using the average spatial distance between two backbone monomers, i and j (Fig 1B). Usually, this metric is denoted as R(s), where s is the number of monomers separating monomers, i and j, or s=|i−j|. In equilibrium melts or globules, chains typically follow R(s) ∝ s^1/2^ (random walk statistics) [71], and single rod-like segments follow R(s) ∝ s. On a small scale, regardless of DP, all RHPs exhibit rod-like chain conformations (R ∝ s) due to the high characteristic ratio ($C_{\infty }$) of PMMA. On a larger scale, 200mer RHPs first plateau and then transition to random walk statistics (R ∝ s^1/2^). However, this transition from rod-like to random walk at s≈50 is not observed in 100mer RHPs. The asphericity indicates that 100mer RHPs are nearly isotropic spheres compared to 200mer RHPs (Fig 1C). The plateau at R(s) ≈ 20 Å for 100mer RHPs suggests that these spherical globules have a R_g_ around 20 Å (by analyzing the snapshots directly, R_g_ = 16.2±0.7 Å). In contrast, a hypothetical spherical 200mers would have a lower surface area-to-volume ratio (∝ 1/r, where r is the radius of the sphere) and may not sufficiently expose and hydrate all negatively charged SPMA monomers on the globule surface, which have a kosmotropic nature [72]. This limitation promotes an ellipsoidal morphology that increaes surface area, facilitating the hydration of kosmotropic SPMA monomers (Fig 1E, SPMA).

For shorter chains (DP = 20 and 50), the sizes are comparable to or smaller than the Kuhn length of PMMA (≈ 15.3 Å) [73], which prevents them from fully collapsing into perfect spherical globules, unlike 100mer RHPs (Fig 1B). The density of RHPs (approximated as polymer mass divided by the volume derived from R_g_) initially increases with chain length in the short-chain regime, but decreases at higher DPs. This trend reflects increased monomer-monomer contacts and monomer dehydration in compactified globules (Fig 1E). Monomer-water contacts are replaced by monomer-monomer contacts, primarily between MMA and PEG moiety, due to their favorable Flory-Huggins interaction parameters (χ<0) [74]. This trend is consistent with our previous work on MMA-based RHPs with less OEGMA and more SPMA contents [42]. For longer chains, density decreases. The plateau of 200mer RHPs near s≈ 50, observed between R ∝ s^1^ and R ∝ s^1/2^ regimes, suggests that the morphology of 200mer RHPs is hierarchical. The large-scale ideal chain (R ∝ s^1/2^) consists of effective “monomers” that correspond approximately to rod-like shortmers (close to 100mer, R ∝ s^1^). The decrease in density can be attributed to the asphericity of the 200-mer RHPs, which are unable to hydrate all charged residues in a spherical morphology as discussed before. Therefore, the sphericity and compactness of MMA-based RHPs are optimized at a chain length of approximately 100 monomers.

Hydration frustration, characterized by hydrated hydrophobic groups and dehydrated polar/hydrophilic groups, is universally observed in MMA-based 100mer RHPs [49]. The degree of core hydration can be experimentally determined through solvatochromism [16], which evaluates the polarity of the local microenvironment. Small-angle neutron scattering (SANS) can provide the radial density distribution of selectively deuterated monomers and, in principle, resolve the spatial distribution of all monomer types within a polymer globule, thereby offering insights into hydration-related structural organization. Morphologically, these RHPs exhibit heterogeneous, patchy surfaces [38]. Monomer hydration is quantified using the normalized number of water molecules in the first solvation shell (NFSS). Defined as the relative population of water molecules within the first solvation shell (3.4 Åradius) compared to a fully solvated monomer of the same type, NFSS enables direct comparison between chemically diverse building blocks [75]. NFSS values range from 1 (fully hydrated) to 0 (completely dehydrated). This calculation includes all atoms, including hydrogen. On average, MMA, EHMA, and OEGMA monomers become increasingly dehydrated as the chain length increases. SPMA hydration remains consistent but is statistically more dehydrated in 200mer RHPs than in 100mer RHPs (p-value $<10^{-7}$). The enhanced MMA hydration in 200mer RHPs offsets the dehydration of other monomers types, contributing to increased relative volume and reduced density. Short chains (e.g., 20mer RHPs) remain well-hydrated across all monomer types, particularly EHMA and OEGMA, because they cannot fully compactify into globules. This highlights a critical distinction in terms of monomer hydration frustration: 20mer RHPs, which exhibit semi-frustrated hydrophobic groups, differ fundamentally from longer RHPs that display full hydration frustration [49].

### Effect of positively charged monomer identity on five-component MMA-based single-chain RHPs and RHP dimers

**Fig 2:**
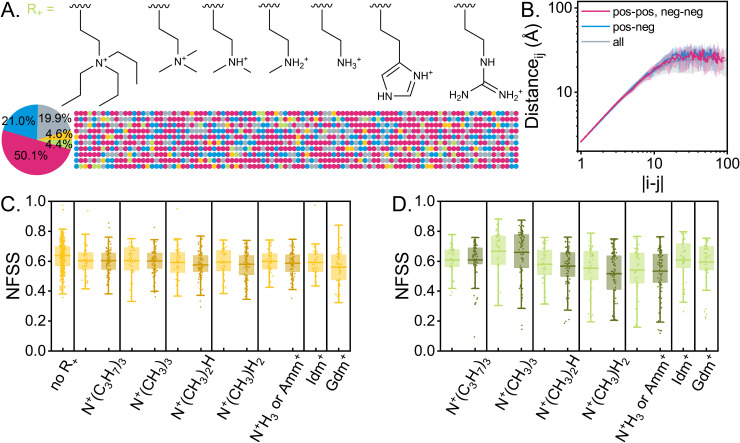
Effect of positively charged monomer identity on five-component MMA-based single-chain RHPs and RHP dimers. (A) The chemical structures of the positively charged monomers (R_+_) in MMA-based RHPs, along with their corresponding sequences and overall five-component composition. Monomers are color-coded as follows: MMA in magenta, OEGMA in blue, EHMA in gray, SPMA in yellow, and R_+_ in green. N = 10 simulations are performed for each R_+_. (B) The spatial distance between monomers *i* and *j* [R(s), s=|i−j|], represented as follows: all monomer pairs (gray), oppositely charged monomers (blue), and like-charged monomers (magenta). Error bars indicate standard deviations across all RHP conformations. NFSS for (C) SPMA and (D) R_+_ in single-chain RHPs (yellow and green symbols) and RHP dimers (brown and olive symbols). The first entry in (C) shows the NFSS of SPMA in the four-component RHPs presented in Fig 1.

To extend the potential functionality of four-component MMA-based RHPs in an experimental setting, we introduce a fifth monomer with a positive charge (R_+_) (Fig 2A). For example, the monomer 2-(dimethylamino)ethyl methacrylate (DMAEMA), with a $-N^{+}(CH_{3}{)}_{2}H$ side chain, has been incorporated to enable temperature-dependent intermolecular interactions for potential use in liquid-liquid phase separation (LLPS) [76] in an experimental setting, as demonstrated in previous work [23]. The degree of methylation decreases from -$N^{+}(CH_{3}{)}_{3}$ to -$N^{+}H_{3}$, resulting in progressively more chaotropic monomers [72]. Other amino acid-based side groups, including guanidinium (-Gdm^+^, analogous to arginine), imidazolium (-Idm^+^, analogous to histidine), and ammonium (-$N^{+}H_{3}$ or -Amm^+^, analogous to lysine), are also incorporated along with a relatively hydrophobic group, -$N^{+}(C_{3}H_{7}{)}_{3}$. R_+_ is fully ionized to isolate the effect of its chemical structure rather than its degree of protonation. Some R_+_ groups, such as imidazole and -$N^{+}(CH_{3}{)}_{2}H$, are weak bases; their partially protonated states are not considered in this work. The composition of the system is adjusted from the original four-component systems to include 50% MMA, 20% EHMA, 20% OEGMA, 5% SPMA, and 5% R_+_ by replacing 5% OEGMA to R_+_, thereby converting the RHPs into polyampholytes. The sequences and overall compositions are shown in Fig 2A.

On a small scale, the scaling behavior of the average spatial distance R(s) between two backbone monomers, i and j, remains consistent with that of four-component RHPs, following R(s) ∝ s for rod-like chain conformations (Fig 2B, gray). The plateau in R(s) occurs around 20 Å, which is similar to that observed in the four-component system. Further analysis of the spatial distance between oppositely charged monomers (blue) and like-charged monomers (magenta) reveals minimal influence of electrostatic attraction or repulsion on either the proximity of oppositely charged monomers or the separation of like-charged monomers. The average spatial distance between charged monomers is unaffected by charge identity, remaining similar to distances between other monomer pairs. This behavior is attributed to the glassy nature of the PMMA backbone [39,58], which hinders chain rearrangement driven by intrachain electrostatic interactions [42,43].

The hydration of SPMA monomers remains consistent across all RHPs, regardless of the chemical structures of R_+_ (Fig 2C, yellow symbols). This consistency highlights the limited influence of intrachain electrostatic interactions on chain rearrangement. Except for Idm+ and Gdm+, the hydration of R_+_ monomers follows the Hofmeister series: more kosmotropic charges (“salting out”, such as -$N^{+}(CH_{3}{)}_{3}$) exhibit higher hydration levels than less kosmotropic charges (“salting in”, such as -$N^{+}H_{3}$) [72] (Fig 2D, green symbols). Interestingly, even the relatively hydrophobic charges (-$N^{+}(C_{3}H_{7}{)}_{3}$) remain well-hydrated, indicating that burying a charged group through brutal thermodynamic forces alone is challenging due to the low dielectric constant of MMA-based RHPs and their alkyl side chains. Additionally, relatively dehydrated charges tend to form complexes with PEG side chains, analogous to crown ether-type interactions [49,77].

To examine oppositely charged-pair interactions, single-chain systems (yellow and green symbols) are extended to dimer systems (brown and olive symbols), each initialized with two identical single-chain conformations (Fig 2C-D). In these setups, one randomly selected pair of oppositely charged monomers from the two chains is intentionally positioned close to each other to examine charge pair interactions. The hydration of SPMA remains the same in the single-chain and dimer systems, while the hydration of R_+_ decreases. This suggests that dimerization is not driven by charge pairs between SPMA and R_+_. The hydration of EHMA and OEGMA monomers (S2 Fig in S1 File) also decreases alongside the hydration of R_+_, which suggests the dimerization results primarily from hydrophobic contacts as well as PEG-R_+_ interaction. This observation aligns with unpublished data on the dimerization of four-component RHPs, which indicate that interfacial interactions are primarily dominated by hydrophobic effects (EHMA-EHMA contacts) [78]. Side-chain rearrangement governs the remodeling of individual chains in the dimer system, consistent with findings with RHP adsorption at liquid-solid interface [43] rather than liquid-liquid interface [45].

### Effect of backbone architecture and chain-level compositions on RHP compactification, dynamics and hydrogen bond formation

**Fig 3:**
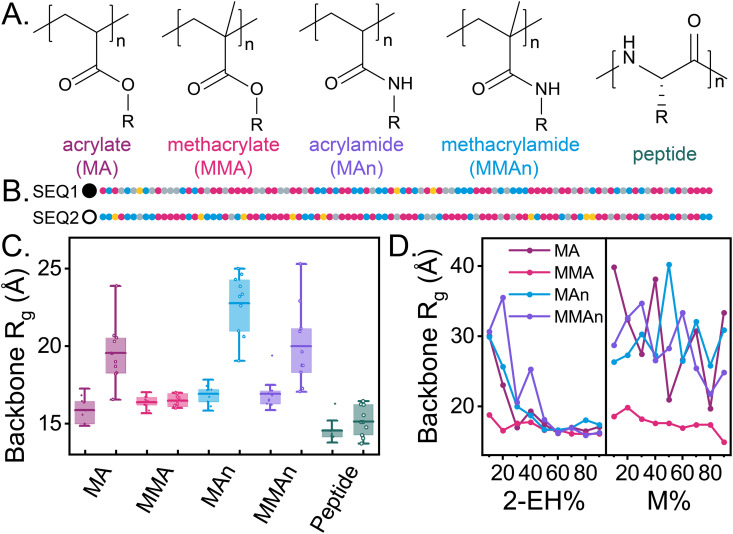
Monomer hydration and backbone R_g_ of peptoid and peptide backbone architecture. The chemical structure of (A) peptoid (N = 10, lignt violet) and (B) peptide (N = 100, olive) backbone architectures. NFSS indicates that both backbone architectures adopt a core-shell morphology.

One-pot synthesis through RAFT copolymerization produces an ensemble of pseudorandom sequences influenced by monomer reactivity ratios [79]. While MMA-based RHPs exhibit sequence insensitivity in terms of compactness, monomer hydration, and protein stabilization potential [50], this property does not extend to other backbone architectures such as acrylate (MA), acrylamide (MAn), or methacrylamide (MMAn). Nature, in contrast, has achieved precise sequence-structure-function relationships in biopolymers like proteins, DNA, and RNA. Efforts to replicate such precise sequence control in synthetic polymers include peptoids (N-substituted glycine backbones) [80], among others sequence-defined systems [81,82]. In this study, to examine backbone effects on the assembly, we investigate peptoid and peptide architectures using the same sequence ensemble (Fig 3). Both architectures collapse into compact globules, with backbone R_g_ of 14.8 ± 0.7 Åfor peptoid- and 14.5 ± 1.1 for peptide-based RHPs. Monomer NFSS indicates a core-shell morphology in both systems, driven by the absence of a negative-*χ* parameter between PEG side groups and the corresponding backbones. These findings demonstrate that without sophisticated sequence design, peptoid- and peptide-based RHPs collapse into compact, though not hydration-frustrated, globules.

**Fig 4:**
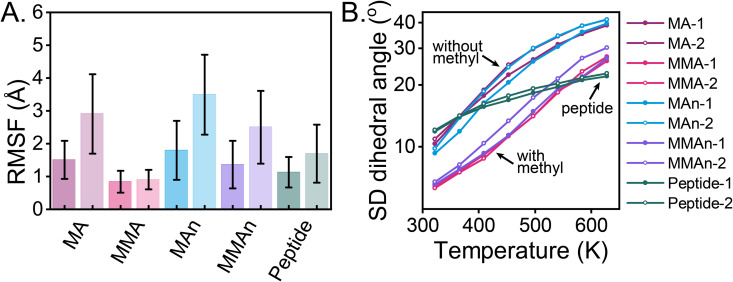
Effect of backbone architecture and chain-level compositions on RHP compactification. (A) The chemical structures of different backbone architectures in four-component RHPs, with backbones color-coded as follows: acrylate (“MA”) in dark red, methacrylate (“MMA”) in magenta, acrylamide (“MAn”) in purple, methacrylamide (“MMAn”) in blue, and peptide in green. (B) Two selected sequences with different chain-level compositions from the same sequence ensemble: one with the smallest (SEQ1, filled symbols) and the other with the largest (SEQ2, open symbols) R_g_ of backbone atoms. Side chains are color-coded following Fig 1A as follows: M in magenta, OEG in blue, 2-EH in gray, and 3-SP in yellow. (C) Backbone R_g_ for the two selected sequences across different backbone architectures (N = 10 for each category). The left entry corresponds to SEQ1, and the right entry corresponds to SEQ2. (D) Backbone R_g_ of random copolymers with two types of monomers: OEG and 2-EH (left panel), and OEG and M (right panel), plotted against the corresponding composition of non-methyl side chains.

We expand the scope of backbone architectures to include soluble variants (Fig 4A). Two four-component sequences with distinct chain-level compositions are selected: SEQ1, a relatively more hydrophobic sequence with 44 MMAs (−6), 27 EHMAs (+7), 26 OEGMAs (+1), and 3 SPMAs (−2), and SEQ2, a relatively more charged sequence with 51 MMAs (+1), 14 EHMAs (−7), 27 OEGMAs (+2), and 8 SPMAs (+3). The numbers in parentheses represent the differences between the actual composition and the targeted composition. The sequence schematics are shown in Fig 4B and corresponding chain-level compositions are shown in Fig 7A.

In MA-, MAn-, and MMAn-based RHPs, SEQ2 (open symbols) adopts a less compact morphology compared to SEQ1 (filled symbols) (Fig 4C). SEQ1 forms compact globules (R_g_ < 20 Å), characterized by a core-shell morphology. In these structures, hydrophobic groups form a core, while polar and charged groups extend into water, a phenomenon known as hydrophobic collapse [49]. In contrast, SEQ2 forms extended coil morphology in these backbone architectures. However, both SEQ1 and SEQ2 exhibit similar compactness in MMA- and peptide-based RHPs, which both feature insoluble backbones. Peptide-based RHPs, like MMA-RHPs, demonstrate sequence insensitivity in globule compactness (see also Fig 3B). The key distinction between these systems lies in the degree of hydration frustration.

The influence of chain-level composition and hydrophobic collapse is also observed in random copolymers with soluble backbone architectures, such as poly(OEGX-*r*-EHX) and poly(OEGX-*r*-MX), where X = A (acrylate), An (acrylamide), or MAn (methacrylamide), with MA (methacrylate) serving as a reference (Fig 4B). MMA-based copolymers consistently exhibit compact structures, a property not observed in other backbone architectures. This suggests that MMA-based polymers offer a broad compositional design space for achieving compact and hydration-frustrated globules. In poly(OEGX-*r*-EHX)s with soluble backbone architectures, extended coil morphologies are observed up to 20% 2-EH side chains. Beyond this threshold, increasing 2-EH content induces hydrophobic core formation, compactifying the chains. Conversely, poly(OEGX-*r*-MX)s remain extended coils regardless of composition, lacking the driving forces required for collapse.

**Fig 5:**
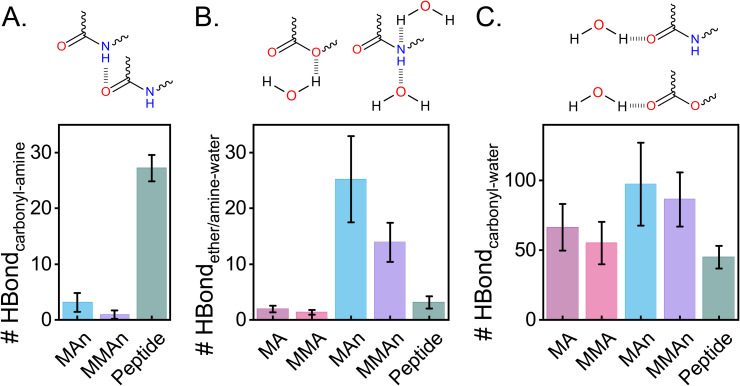
Effect of backbone architectures and chain-level compositions on RHP dynamics. (A) Root-mean-square fluctuation (RMSF) of backbone C*α* atoms and (B) standard deviation of backbone dihedral angles (SD dihedral angle) for two selected sequences across different backbone architectures. For RMSF, results are averaged over ten conformations. The left entry corresponds to SEQ1, and the right entry corresponds to SEQ2. For SD dihedral angles, values are averaged over 5 ns of the annealing process (filled symbols represent SEQ1, and open symbols represent SEQ2).

The dynamics of RHPs are influenced by both backbone architecture and chain-level composition. To quantify their effects, we examine the backbone atomic root-mean-square fluctuation (RMSF) and the standard deviation of backbone dihedral angles (SD dihedral) (Fig 5). SEQ2 exhibits greater mobility than SEQ1 in both metrics, attributable to the extended coil morphology of SEQ2, which increases polymer-water contacts and enhances mobility compared to the globule morphology of SEQ1. Backbones with soluble or peptide architectures are more dynamic than those with MMA backbone architectures. This disparity arises from the glassy nature of PMMA and the additional stabilizing effect of favorable MMA-PEG contacts, the latter of which is absent in peptide-based RHPs due to unfavorable peptide-PEG interactions (χ≥0). The dynamics of MMA-based globules are comparable to those of the same polymer in the melt or vacuum state [39]. Backbone dihedral angle fluctuations are strongly influenced by the degree of backbone methylation. Architectures without methyl groups (e.g., acrylate and acrylamide) exhibit greater mobility than methylated architectures (e.g., methacrylate and methacrylamide) due to steric hindrance introduced by methyl groups [59]. Among all backbone architectures, peptide backbones exhibit the least increase in dihedral angle fluctuations with rising temperature.

The differences in compactness and dynamics between SEQ1 and SEQ2, despite both originating from the same sequence ensemble accessible via one-pot synthesis, highlight the capability of MMA-based RHPs to achieve convergent properties. However, this sequence-insensitive behavior is not observed in other backbone architectures. Experimentally, purification processes such as chromatography can shift the overall composition of MMA-based RHPs [79], and MA-based RHPs have been shown to exhibit distinct subpopulations [83], which are not observed in MMA-based RHPs [84]. Our computational results confirm that the properties of MMA-based RHPs are more homogeneous and less sequence-sensitive, than those of MA-based RHPs [50], reflecting the coexistence of coil and globule conformations.

**Fig 6:**
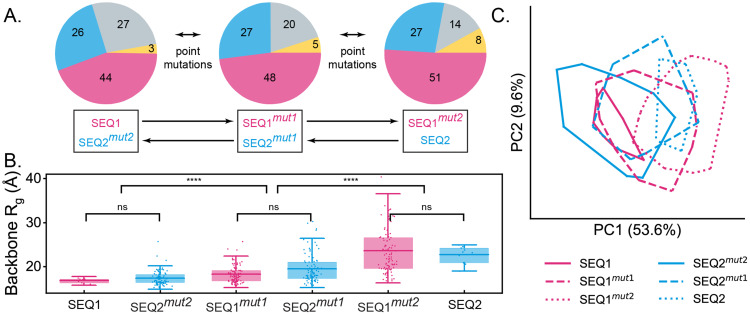
Effect of backbone architectures on hydrogen bond formation. The number of hydrogen bonds in a single RHP chain (DP = 100, with N = 100) is analyzed as follows: (A) between carbonyl and amide groups, (B) between carbonyl groups and water molecules, and (C) between ether/amine groups and water molecules. Schematics are shown, and the results are averaged over 20 conformations from two selected sequences. Error bars indicate standard deviations across all RHP conformations. The color coding of backbone architectures follows Fig 4.

This discrepancy in dynamics across backbone identities can be attributed to the backbone’s ability to form hydrogen bonds, particularly intrachain hydrogen bonds between amine and carbonyl groups, as well as the hydrogen bonds between electronegative backbone atoms and water molecules. Peptide-based RHPs form significantly more intrachain hydrogen bonds (amine-carbonyl interactions) compared to MAn- and MMAn-based RHPs (Fig 6A), which act as a major driving force for compactification in peptide-based RHPs, similar to the behavior observed in proteins. MMA-based RHPs lack such intramolecular hydrogen bonds; their compactification is instead driven by hydrophobic interactions of the backbone and favorable negative-*χ* parameter between MMA and PEG. This highlights the distinct mechanism behind MMA-based RHP compactification, as opposed to peptide-based RHPs that are dominated by hydrogen bond.

Hydrogen bonding also influences backbone-water interactions. The number of hydrogen bonds formed between the backbone (carbonyl/ether/amine groups) and water molecules (Fig 6B-C) correlates well with the backbone fluctuation observed in Fig 5. Increased backbone solubilization through intermolecular hydrogen bonding with water enhances backbone mobility. The significance of hydrogen bonding underscores that the sequence sensitivity in aqueous polymer globules cannot be predicted solely from the glass transition temperature (T_g_) of the homopolymer melts (e.g., polyacrylate, polymethacrylate, polyacrylamide, and polymethacrylamide) (S3 Fig, data adapted from [59]). Melt systems fail to capture polymer-water interactions, while aqueous globule systems place less emphasis on the role of intermonomer hydrogen bonding. Both factors are essential for accurately predicting the behavior of RHPs in aqueous environments.

### Evolution of chain-level compositions in SEQ1 and SEQ2 of MAn-based RHPs, and sequence sensitivity assessment

To examine the effect of chain-level composition on compactness and monomer hydration patterns, we employ a two-step evolution process in MAn-based RHPs. Compactness is quantified by backbone R_g_, and monomer hydration patterns are characterized using the hydration array introduced in our recent work [50]. This hydration array is a 73-dimensional array, representing the NFSS values for four monomer types (42 M, 14 2-EH, 14 OEG, and 3 3-SP) that are shared across all sequences. The similarity between systems is assessed by comparing the overlap of convex hulls formed by dimensionally-reduced hydration arrays using PCA (See Methods for details).

**Fig 7:**
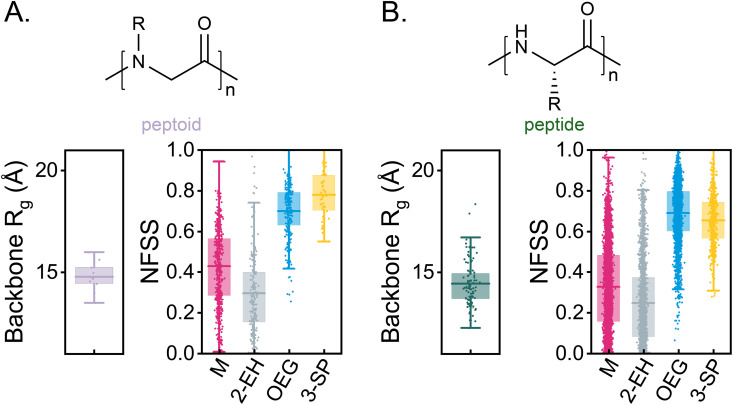
Evolution of chain-level compositions in SEQ1 and SEQ2 of MAn-based RHPs, and sequence sensitivity assessment. (A) Two-step chain-level composition evolution. The evolution process involves an intermediate composition, illustrated using pie charts. Monomers are color-coded as follows: MAn in magenta, OEGAn in blue, EHAn in gray, and SPAn in yellow. In each step, monomers in the selected sequence are randomly mutated to generate ten offspring sequences through point mutations that progressively match the target composition. (B) Backbone R_g_ values of six systems. Statistical significance is evaluated using a two-sample t-test, with p-values denoted as follows: ns (p ≥ 0.05) and **** (p < 0.0005). (C) Convex hulls of six systems in monomer hydration patterns via principal components analysis. Convex hulls illustrate monomer hydration patterns of the same system, with hulls of the same color indicating high sequence similarity arising from to point mutation evolution and hulls with the same patterns representing identical chain-level compositions. The variance explained by the first and second principal components is 53.6% and 9.6%.

The chain-level composition evolution of SEQ1 and SEQ2 proceeds in two steps, passing through a shared intermediate composition that represents the average composition of SEQ1 and SEQ2. The compositions of SEQ1, SEQ2, and the intermediate are shown in Fig 7A. In each step, the selected sequence undergoes random point mutations to generate ten new sequences that match the targeted composition. For example, in SEQ1, seven EHAn monomers are randomly mutated into one OEGAn, four MAn, and two SPAn monomers, producing ten sequences collectively referred to as SEQ1^mut1^, which correspond to the intermediate composition. Subsequently, six EHAn monomers in SEQ1^mut1^ are further mutated into three MAn and three SPAn, generating ten sequences labeled SEQ1^mut2^, which match the chain-level composition of SEQ2. Conversely, SEQ2 is first mutated to generate SEQ2^mut1^, matching the intermediate composition, and then further mutated to generate SEQ2^mut2^, which aligns with the chain-level composition of SEQ1. Full sequence schematics for these steps are shown in S4 Fig in S1 File. As a brief note, SEQ1 and SEQ2^mut2^, SEQ1^mut1^ and SEQ2^mut1^, and SEQ2 and SEQ1^mut2^ share the same chain-level composition. Additionally, SEQ1, SEQ1^mut1^, and SEQ1^mut2^ exhibit high sequence similarity, as do SEQ2, SEQ2^mut1^, and SEQ2^mut2^.

From SEQ1 to SEQ1^mut2^, the backbone R_g_ increases, indicating decompactification. Conversely, from SEQ2 to SEQ2^mut2^, the backbone R_g_ decreases, reflecting compactification. Moreover, systems with the same chain-level compositions (SEQ1 and SEQ2^mut2^, SEQ1^mut1^ and SEQ2^mut1^, and SEQ2 and SEQ1^mut2^) exhibit nearly identical backbone R_g_ distributions. These findings demonstrate that compactness in MAn-based RHPs is primarily dictated by chain-level composition, rather than specific sequence details.

The convex hulls formed by SEQ1 (solid magenta, left) and SEQ2 (dotted blue, right) do not overlap. During the evolutionary process, the convex hull for SEQ1 shifts to the right as it evolves into SEQ1^mut2^, while the convex hull for SEQ2 shifts to the left as it transitions into SEQ2^mut2^. Convex hulls corresponding to systems with the same chain-level composition but low sequence similarity overlap. Notably, convex hulls for systems with different chain-level compositions and sequences (e.g., SEQ1^mut2^ and SEQ2^mut2^) also show significant overlap. These observations suggest that monomer hydration patterns are governed by a combined influence between sequence identity and chain-level composition, rather than by either factor alone in MAn-based RHPs. In contrast, MMA-based RHPs generally exhibit more robust properties with respect to sequence and chain-level composition, even when generated from the same sequence ensemble obtained through stochastic synthesis.

### Effect of the length of PEG pendant group and side-chain micropolarity in MMAn-based RHPs

**Fig 8:**
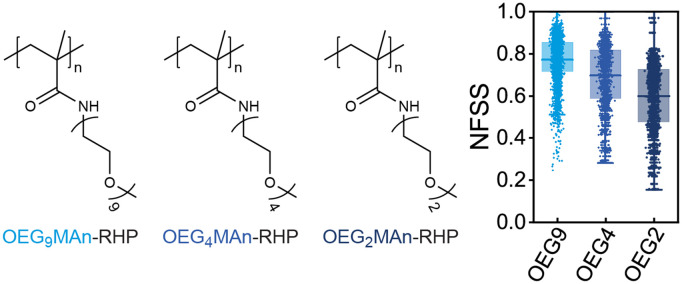
Effect of the length of PEG pendant group of OEGMAn in MMAn-based RHPs. The chemical structures of OEGMAn with nine (OEG_9_MAn), four (OEG_4_MAn), and two (OEG_2_MAn) PEG repeating units, and their corresponding NFSS values. Each system is simulated for N = 100 simulations.

The water solubility of poly(OEG_n_MA) is heavily dependent on the length of its oligo(ethylene glycol) (PEG) side chains units, particularly in low-n regime [85,86]. For example, poly(OEG_n_MA) is insoluble when n = 1, while reversible cloud points occur at 26 ^°^C and 52 ^°^C for n = 2 and n = 3, respectively, demonstrating thermoresponsive behavior. In this study, we examine the solubility of OEG_n_MAn monomers within MMAn-based RHPs (Fig 8). The hydration of the monomers, quantified by NFSS, decreases significantly as the PEG pendant group length shortens from 9 to 4–2 repeat units, with hydration values dropping from 0.77 to 0.60. Atomic partial charges calculated via RESP reveal that the terminal and penultimate oxygens in the polyether chain (−0.466 and −0.593, respectively) carry smaller magnitudes of partial charge compared to the other oxygens (−0.682). This difference in charge distribution increases the hydrophobicity of the monomer as the PEG repeat unit number decreases. Such reductions in hydration and increased hydrophobicity with shorter PEG side chains are consistent with trends observed in other polyethers, where solubility is strongly linked to chain length and partial charge distributions [87]. These findings emphasize the critical role of PEG chain length in determining the solubility and hydration behavior of MMAn-based RHPs.

**Fig 9:**
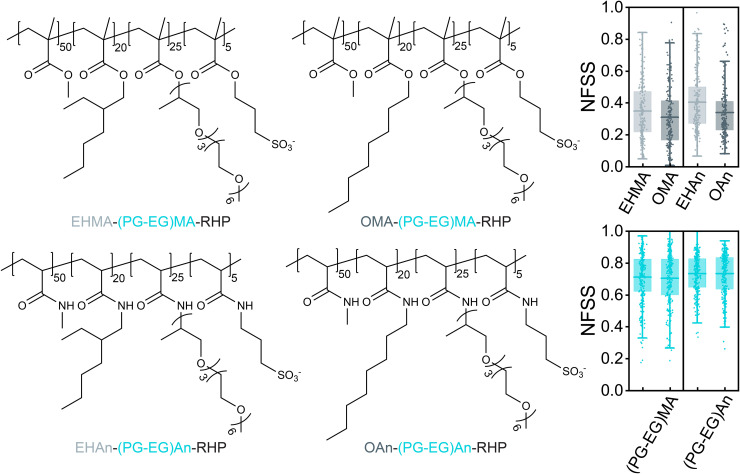
Effect of the side chain micropolarity in MMAn- and MAn-based RHPs. The chemical structure of RHPs with linear (O-) and branched (EH-) alkyl side chains, and Jeffamine-like (PG-EG), and their corresponding NFSS values. Each system is simulated for N = 10 simulations. In both panels, the left entry corresponds to the EH- side chain, and the right entry corresponds to the O- side chain for each backbone architecture.

The polarity of alkyl side chains, including isobutyl, 2-ethylhexyl, and octyl, has been shown to influence their hydration patterns, ranging from frustrated isobutyl and 2-ethylhexyl, to non-frustrated octyl [49]. This difference in micropolarity, such as between valine, alanine, and glycine, is known to govern the organization of elastin-like polypeptide (ELP) condensates [88]. Here, we further investigate the hydration patterns between 2-ethylhexyl and octyl, as well as between poly(ethylene glycol) (PEG) and poly(propylene glycol-co-ethylene glycol) (PG-EG) side chains (Fig 9). The latter polyether has been used in SCNP to modulate both water solubility [89,90] and compactness [91]. The branched 2-ethylhexyl side chain and polyether (PG-EG) exhibit greater hydration than their linear counterparts, octyl and PEG. The higher degree of branching promotes hydration, which can be attributed to the polar carbon atoms in the branching. For example, the average atomic partial charges of the terminal atoms in polypropylene glycol is −0.404, compared to −0.303 in polyethylene glycol. This increased hydration of the polar group in EHMA-(PG-EG)MA-RHP and OMA-(PG-EG)MA-RHP results in hydration patterns characterized as semi-frustrated (frustrated hydrophobic group but non-frustrated polar group) and core-shell (non-frustrated hydrophobic and polar groups), in comparison with the fully-frustrated hydration pattern observed in EHMA-OEGMA-RHP.

## Conclusion

This study offers a detailed exploration of the factors that govern the compactness, dynamics, hydration, and solubility of RHPs, focusing on variations in chain length, monomer identity, backbone architecture, and chain-level composition. By systematically examining four- and five-component MMA-based RHPs alongside alternative backbone types and side chains with distinct micropolarities, we uncover molecular principles underlying their assembly and hydration behavior. Together, these findings underscore the tunable nature of RHPs and demonstrate the expansive design space through MMA-based RHPs. Such polymers hold significant promise as synthetic analogs of proteins, enabling the rational design of protein-mimetic materials with customizable structure and function.

We would like to highlight that for MMA-based RHPs, which have demonstrated versatile protein-like functions experimentally, a chain length of approximately 100 monomers appears to be optimal for achieving both compactness and balanced hydration. The negative *χ* parameter between MMA and PEG gives rise to a distinct form of hydration frustration that sets MMA-based RHPs apart from other backbone architectures and imparts sequence insensitivity to many of their physicochemical properties.

Future studies can expand the applicability of RHPs by exploring the effects of multivalent ions and cofactor binding, which could facilitate molecular encapsulation and open new opportunities for catalytic applications and delivery vehicles. Temperature- and pH-dependent transitions will further enable the use of RHPs as responsive materials. Coupled with autonomous laboratory [28,36,37], advanced experimental and computational techniques will support the rational design and engineering of RHPs with protein-like functions, ultimately unlocking their full potential for diverse applications [19].

## Supporting information

S1 File Supplementary information (S1–S4 Fig). (PDF)
