# Supplementary material for: Computational design of functional random heteropolymers through atomistic simulations
Source: PLoS One. 2026 Mar 18;21(3):e0343799. doi: 10.1371/journal.pone.0343799 (PMC12998813; doi:10.1371/journal.pone.0343799)
Supplement: S1 File — (PDF) [file pone.0343799.s001.pdf]

Supporting Information: Computational design of functional random heteropolymers through atomistic simulations

Tianyi Jin,[†,‡] Collin S. Lung,[†] Ting Xu,[¶,§,∥,⊥] Connor W. Coley,[†,#] and Alfredo Alexander-Katz[*,‡]

†*Department of Chemical Engineering, Massachusetts Institute of Technology, Cambridge, MA, USA*

‡*Department of Materials Science and Engineering, Massachusetts Institute of Technology, Cambridge, MA, USA*

¶*Department of Materials Science and Engineering, University of California, Berkeley, Berkeley, CA, USA*

§*Department of Chemistry, University of California, Berkeley, Berkeley, CA, USA*

∥*Materials Science Division, Lawrence Berkeley National Laboratory, Berkeley, CA, USA*

⊥*Kavli Energy NanoScience Institute, Berkeley, CA, USA*

#*Department of Electrical Engineering and Computer Science, Massachusetts Institute of Technology, Cambridge, MA, USA*

E-mail: aalexand@mit.edu

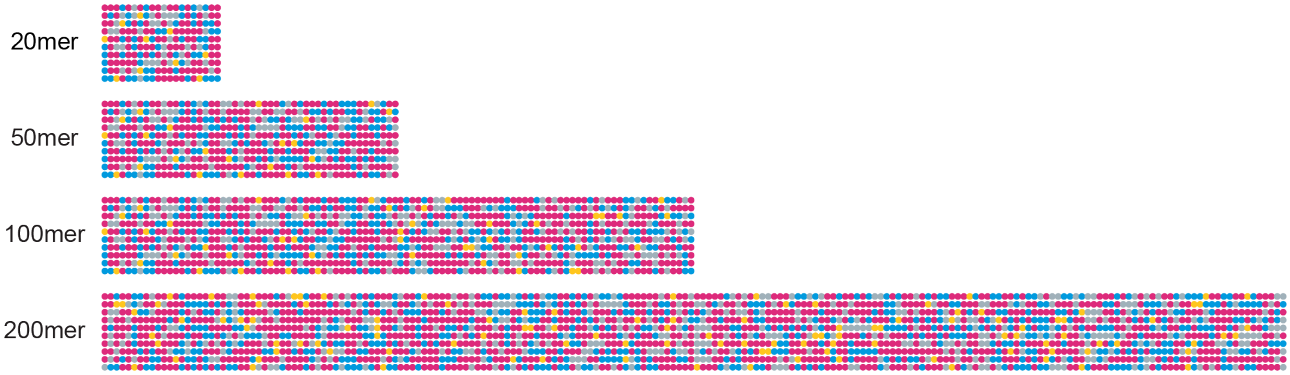

S1 Fig: Sequence schematics of 20mer, 50mer,[1] 100mer[2] and 200mer four-component RHPs. Monomers are color-coded as follows: MMA in magenta, OEGMA in blue, EHMA in gray, and SPMA in yellow.

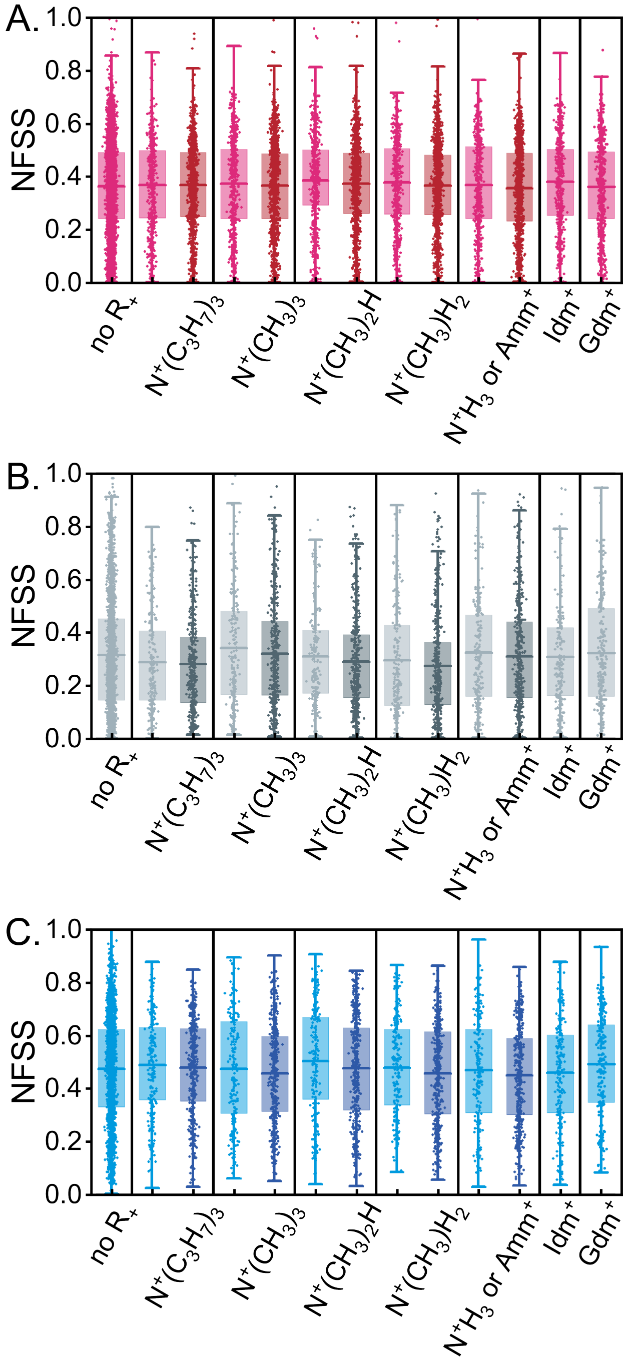

S2 Fig: The normalized first solvation shell (NFSS) for (A) MMA, (B) EHMA, and (C) OEGMA in single-chain RHPs (left columns) and RHP dimers (right columns). The first columns show the NFSS in the four-component RHPs presented in Fig 1.

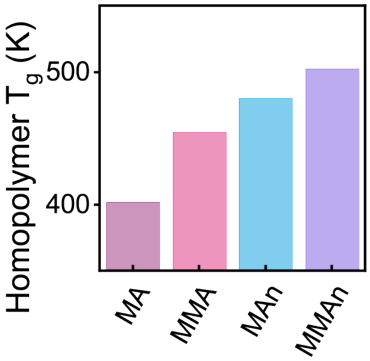

S3 Fig: Glass transition temperatures (T_g) of homopolymers: poly(methyl acrylate) (MA), poly(methyl methacrylate) (MMA), poly(methyl acrylamide) (MAn) and poly(methyl methacrylamide) (MMAn).[3]

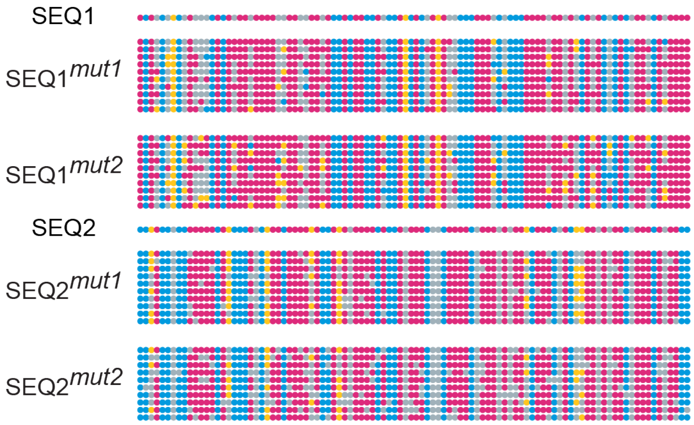

S4 Fig: Sequence schematics of SEQ1, SEQ1^{mut1}, SEQ1^{mut2}, SEQ2, SEQ2^{mut1} and SEQ2^{mut2}. Monomers are color-coded as follows: MAn in magenta, OEGAn in blue, EHAn in gray, and SPAn in yellow.

References

(1) Jin, T.; Hilburg, S. L.; Alexander-Katz, A. Glass transition of random heteropolymers: A molecular dynamics simulation study in melt, in water, and in vacuum. *Polymer* **2023**, *265*, 125503.

(2) Hilburg, S. L.; Ruan, Z.; Xu, T.; Alexander-Katz, A. Behavior of Protein-Inspired Synthetic Random Heteropolymers. *Macromolecules* **2020**, *53*, 9187–9199.

(3) Jin, T.; Coley, C. W.; Alexander-Katz, A. A Computationally Informed Unified View on the Effect of Polarity and Sterics on the Glass Transition in Vinyl-based Polymer Melts. *ACS Macro Letters* **2023**, *12*, 1517–1522.
